# Persistent up-regulation of polyribosomes at synapses during long-term memory, reconsolidation, and extinction of associative memory

**DOI:** 10.1101/lm.053577.122

**Published:** 2022-08

**Authors:** Linnaea E. Ostroff, Christopher K. Cain

**Affiliations:** 1 Department of Physiology and Neurobiology, University of Connecticut, Storrs, Connecticut 06269, USA; 2 Connecticut Institute for the Brain and Cognitive Science, University of Connecticut, Storrs, Connecticut 06269, USA; 3 Institute of Materials Science, University of Connecticut, Storrs, Connecticut 06269, USA; 4 Emotional Brain Institute, Nathan Kline Institute for Psychiatric Research, Orangeburg, New York 10962, USA; 5 Child and Adolescent Psychiatry, New York University Langone Health, New York, New York 10016, USA

## Abstract

Local protein synthesis at synapses can provide a rapid supply of proteins to support synaptic changes during consolidation of new memories, but its role in the maintenance or updating of established memories is unknown. Consolidation requires new protein synthesis in the period immediately following learning, whereas established memories are resistant to protein synthesis inhibitors. We have previously reported that polyribosomes are up-regulated in the lateral amygdala (LA) during consolidation of aversive-cued Pavlovian conditioning. In this study, we used serial section electron microscopy reconstructions to determine whether the distribution of dendritic polyribosomes returns to baseline during the long-term memory phase. Relative to control groups, long-term memory was associated with up-regulation of polyribosomes throughout dendrites, including in dendritic spines of all sizes. Retrieval of a consolidated memory by presentation of a small number of cues induces a new, transient requirement for protein synthesis to maintain the memory, while presentation of a large number of cues results in extinction learning, forming a new memory. One hour after retrieval or extinction training, the distribution of dendritic polyribosomes was similar except in the smallest spines, which had more polyribosomes in the extinction group. Our results demonstrate that the effects of learning on dendritic polyribosomes are not restricted to the transient translation-dependent phase of memory formation. Cued Pavlovian conditioning induces persistent synapse strengthening in the LA that is not reversed by retrieval or extinction, and dendritic polyribosomes may therefore correlate generally with synapse strength as opposed to recent activity or transient translational processes.

The formation of long-term memory involves a consolidation phase in the period immediately after learning, during which new proteins are required to stabilize learning-induced synapse remodeling (Davis and Squire 1984; Mayford et al. 2012; Rosenberg et al. 2014; Segal 2017). There is evidence that local protein synthesis in dendrites is essential for consolidation of long-term memory and related forms of synaptic plasticity (Holt and Schuman 2013), but its exact role is not well understood. Dendritic translation can supply new proteins to synapses rapidly, and potentially with synapse-specific spatial precision. Thousands of mRNAs have been identified in dendrites, many of which encode synaptic proteins (Poon et al. 2006; Zhong et al. 2006; Cajigas et al. 2012; Tushev et al. 2018; Middleton et al. 2019), and mRNA is present in dendritic spines (Tiruchinapalli et al. 2003; Hafner et al. 2019). The ability of dendritic mRNAs to remain dormant until they are unmasked by synaptic activity (Doyle and Kiebler 2011; Buxbaum et al. 2014; Hutten et al. 2014) provides a mechanism for rapid and targeted translation at synapses. Synaptic activity during learning triggers a transient up-regulation of new synaptic proteins in dendrites (Redondo and Morris 2011; Moncada et al. 2015), and the spatiotemporal constraints on these new proteins strongly suggest that they are translated locally (Sajikumar et al. 2007; Doyle and Kiebler 2011). We have previously found by serial section transmission electron microscopy (ssTEM) volume reconstruction that polyribosomes and translation factors are up-regulated in dendritic spines in the rat lateral amygdala (LA) 1 h after cued aversive Pavlovian conditioning (Ostroff et al. 2010, 2017; Gindina et al. 2021). These polyribosomes presumably represent translation supporting consolidation, but no studies have addressed whether dendritic translation remains elevated or returns to baseline in the long-term memory phase.

Cued aversive Pavlovian conditioning, also referred to as fear or threat conditioning, is an extensively studied learning paradigm in which a sensory cue is paired with an unpleasant stimulus—typically an auditory cue with a mild shock—to create an associative memory between the two (LeDoux 2000; Maren 2001). There is strong evidence that this memory is mediated by protein synthesis-dependent strengthening of LA synapses during a short window after learning. Enhanced synaptic transmission is observed in the LA after conditioning (McKernan and Shinnick-Gallagher 1997; Rogan et al. 1997; Sah et al. 2008), and consolidation requires protein synthesis in the LA immediately after training, but not 6 or 24 h later (Nader et al. 2000; Schafe and LeDoux 2000; Maren et al. 2003). The extracellular signal-regulated/mitogen-activated protein kinase (ERK/MAPK), which regulates translation (Kelleher et al. 2004), is transiently phosphorylated in the LA 1 h after learning, and this phosphorylation is required for both memory consolidation (Schafe et al. 2000) and synaptic plasticity in the LA (Huang et al. 2000; Schafe et al. 2008).

Although dormant long-term memories are stable, retrieval induces a new labile phase called reconsolidation, during which the memory can be updated, weakened, or strengthened (Dudai 2012). As in consolidation, postretrieval inhibition of protein synthesis or ERK/MAPK phosphorylation in the LA impairs reconsolidation of the memory and associated synaptic plasticity (Nader et al. 2000; Duvarci et al. 2005; Doyere et al. 2007). A transient supply of necessary new proteins is available to synapses during reconsolidation (Orlandi et al. 2020), but whether these proteins are synthesized in dendrites is unknown. Both consolidation and reconsolidation are impaired by broad protein synthesis inhibitors, and there is substantial evidence that consolidation requires translation initiation, the step in which polyribosomes are formed (Gkogkas et al. 2010; Santini et al. 2014). Interestingly, one study found that inhibition of the predominant initiation process impaired consolidation but not reconsolidation, suggesting that the role of translation differs between the two processes (Hoeffer et al. 2011). Since polyribosomes can be stalled for later reactivation (Richter and Coller 2015), reconsolidation could rely on translation of pre-existing polyribosomes.

Reconsolidation is triggered by a small number of retrieval cues, but retrieval with a large number of cues induces extinction learning, in which the cue loses its ability to elicit defensive responses (Myers and Davis 2007). There is ample evidence that plasticity important for extinction occurs in the basolateral amygdala (BLA; which includes the LA), though it is unclear exactly how this relates to the original memory trace in the dorsal LA (Bouton et al. 2021). For instance, consolidation of extinction is impaired by pretraining systemic inhibition of protein synthesis (Suzuki et al. 2004) and by pretraining inhibition of protein synthesis or ERK/MAPK in the BLA (Lin et al. 2003c; Herry et al. 2006). However, the Lin et al. (2003c) study measured the effects of protein synthesis inhibition in the BLA 30 min after extinction training, which is typically thought to reflect short-term memory. Subsequent work by another group found that postextinction training inhibition of protein synthesis impaired reconsolidation, making it difficult to assess the effects on extinction consolidation (Duvarci et al. 2006). There are also ongoing debates about the relative contribution of “erasure” versus “new learning” processes in extinction. Evidence that protein synthesis-dependent depotentiation of CS inputs to the LA contributes to extinction suggests up-regulation of polyribosomes in the LA pyramidal cells storing the original trace (Lin et al. 2003a,b,c; Kim et al. 2009). However, up-regulation of polyribosomes is also possible if extinction plasticity occurs in other cells or regions of the brain, as repeated retrieval trials may strongly trigger reconsolidation processes. Complicating things further, it appears that extinction can halt reconsolidation (Suzuki et al. 2004).

To investigate the dynamics of local translation in the context of an established memory, we used ssTEM to quantify dendritic polyribosome distribution in the LA during the long-term memory phase of Pavlovian conditioning, reconsolidation, and consolidation of extinction. We hypothesized that polyribosomes would not be up-regulated in the long-term memory condition relative to controls, since memory maintenance is resistant to protein synthesis inhibition at this time point. We also hypothesized that both retrieval and extinction would induce up-regulation of polyribosomes, but in different patterns; for example, reconsolidation processes could be reflected in polyribosomes near large synapses, but extinction could result in loss of these synapses and perhaps more generalized polyribosome distribution.

## Results

### Behavior

**Figure 1.**
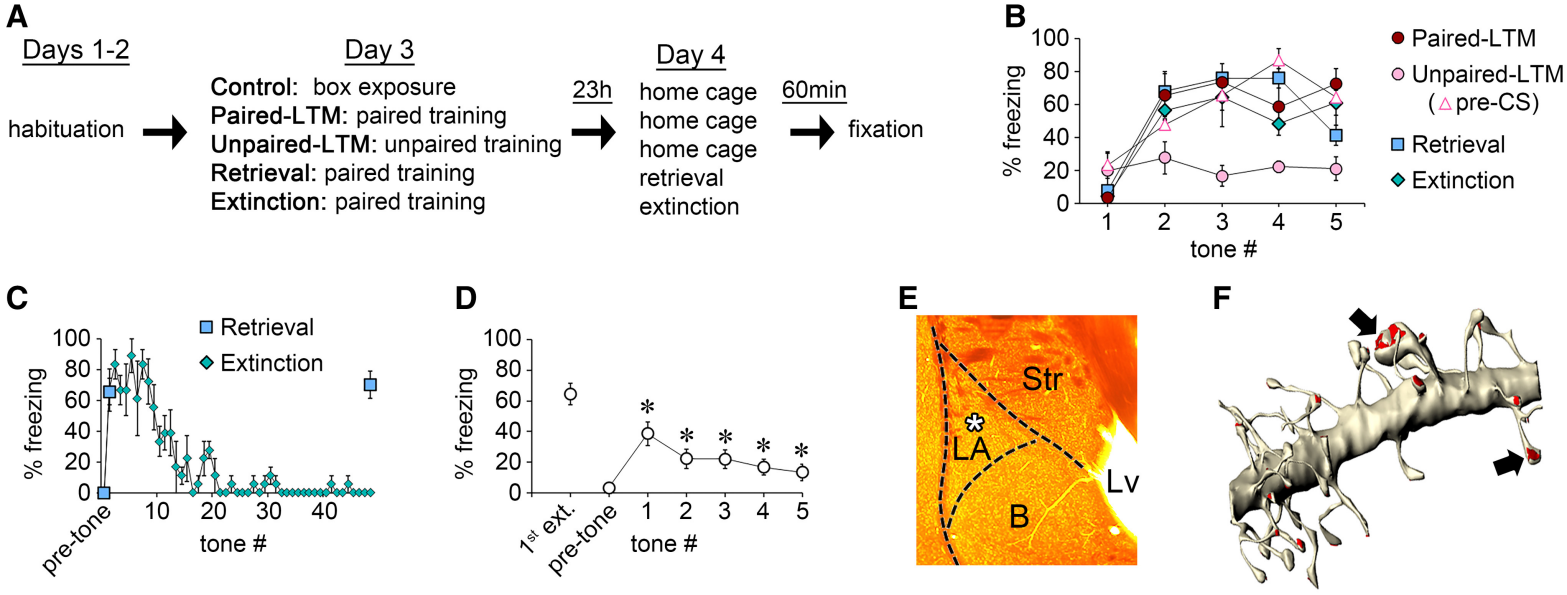
Experimental design and methods. (*A*) Experimental workflow. (*B*) Freezing to each of the five tones during training on day 3, and during the pretone period for the unpaired-LTM group. The subjects used for EM analysis in the paired-LTM, unpaired-LTM, retrieval, and extinction groups are shown. In the unpaired-LTM group, freezing was significantly lower during the fourth and fifth tones relative to the 30-sec pretone period (tone 4: *t*_(3)_ = 8.04, *P* = 0.015; tone 5: *t*_(3)_ = 4.91, *P* = 0.039). (*C*) Plot showing freezing during each of the 48 tones presented to the extinction group and the two tones presented to the retrieval group on day 4. (*D*) Long-term memory test 24 h after extinction training in a separate group of rats (*n* = 16). Freezing to the first tone during extinction training was significantly higher than freezing to each of the five test tones (paired, two-tailed *t*-tests: tone 1: *t*_(15)_ = 5.97, *P* < 0.0001; tone 2: *t*_(15)_ = 8.38, *P* < 0.00001; tone 3: *t*_(15)_ = 8.72, *P* < 0.00001; tone 4: *t*_(15)_ = 10.69, *P* < 0.00001; tone 5: *t*_(15)_ = 8.57, *P* < 0.00001). (*E*) Section prepared for EM from one of the paired-LTM subjects. The white asterisk indicates the area sampled for EM reconstruction. (LA) Lateral amygdala, (B) basal amygdala, (Str) striatum, (Lv) lateral ventricle. (*F*) Reconstructed spiny dendrite (length = 9 µm) from the same sample. Arrows indicate examples of spine synapses.

Five different training protocols were used to compare dendrites during the long-term memory phase (Fig. 1A). After 2 d of habituation to the training chamber, three groups of adult male rats were presented with auditory tones paired with footshocks (the paired-LTM, retrieval, and extinction groups), a fourth group was presented with unpaired tones and shocks (the unpaired-LTM group), and a fifth group was placed in the training chamber but given no tones or shocks (the control group). The three groups given paired training developed increasingly high levels of freezing to the tone over the course of the training session, while unpaired training resulted in freezing during the pretone period with lower freezing to the tone itself (Fig. 1B). In a previous study using the same paired and unpaired training protocols, we found that only the paired protocol produced robust freezing to the tone when tested 24 h after training in a novel chamber (Ostroff et al. 2010). The control, unpaired-LTM, and paired-LTM groups remained in their home cages with no further behavioral manipulations until brain collection 24 h after training. On the fourth day, the retrieval and extinction groups were placed in a novel chamber, and the extinction group was presented with 48 tones while the retrieval group was presented with two tones timed to match the first and last tones of the extinction group. Both groups froze to the first tone, but by the final tone freezing was abolished in the extinction group only (Fig. 1C). To verify that the 48-tone protocol produced long-term memory for extinction, a separate group of rats was trained and tested in the extinction context 24 h later. Freezing to five test tones was significantly lower than to the first tone of extinction (Fig. 1D). Together, these data demonstrate robust acquisition and LTM using our paired conditioning and extinction protocols. Unpaired rats showed no increase in cue-elicited freezing. Retrieval rats showed no reduction of freezing with time and provided a comparison with extinction rats that differed only in the amount of cue exposure received on day 4.

All rats were perfused with fixative exactly 24 h after the first tone of paired or unpaired training, which was also 1 h after the first tone of retrieval or extinction. In previous ssTEM studies of consolidation, we collected samples 1 h after the first tone of paired and unpaired training (Ostroff et al. 2010, 2017). Thus, the paired-LTM, unpaired-LTM, and control group samples differed from those in the earlier data sets only in that they were collected in the long-term memory phase instead of during consolidation, whereas the retrieval and extinction samples were collected at the same point during either reconsolidation or consolidation of extinction. Tissue sections at a mid-caudal level of the left LA were prepared for EM, and ssTEM image volumes were acquired from an area at the center of the dorsolateral subdivision of the LA (Fig. 1E). This dorsal region of the basolateral amygdala receives sensory inputs from the auditory thalamus and cortex that have been implicated in cued, but not contextual, fear conditioning (Sigurdsson et al. 2007). Changes related to contextual conditioning in all groups were not expected, as hippocampal inputs implicated in contextual conditioning innervate ventral regions of the basolateral amygdala (Kim and Cho 2020). Spiny dendritic segments (Fig. 1F) presumably belonging to excitatory projection neurons (McDonald 1982, 1992) were chosen for analysis, while large apical dendrites and aspinous dendrites from putative inhibitory neurons (McDonald and Pearson 1989) were excluded. We chose to focus our analysis on a critical region of the LA known to be required for the learning, consolidation, performance, and reconsolidation of threat conditioning plasticity (Cain et al. 2008). Extinction also depends on plasticity in the basolateral amygdala (for review, see Bouton et al. 2021), most clearly in studies implicating depotentiation synapses strengthened during threat conditioning (Lin et al. 2003a,b,c; Kim et al. 2009). Although critical extinction plasticity and consolidation processes occur in other brain regions (e.g., infralimbic cortex) and cell types (e.g., intercalated cells) (Milad and Quirk 2012; Duvarci and Pare 2014), the highly resource- and labor-intensive nature of ssTEM limits the number of samples that can be examined, so in this study we focused specifically on the structural correlates of a long-term memory in its latent state and after retrieval.

### Up-regulation of dendritic polyribosomes during long-term memory

**Figure 2.**
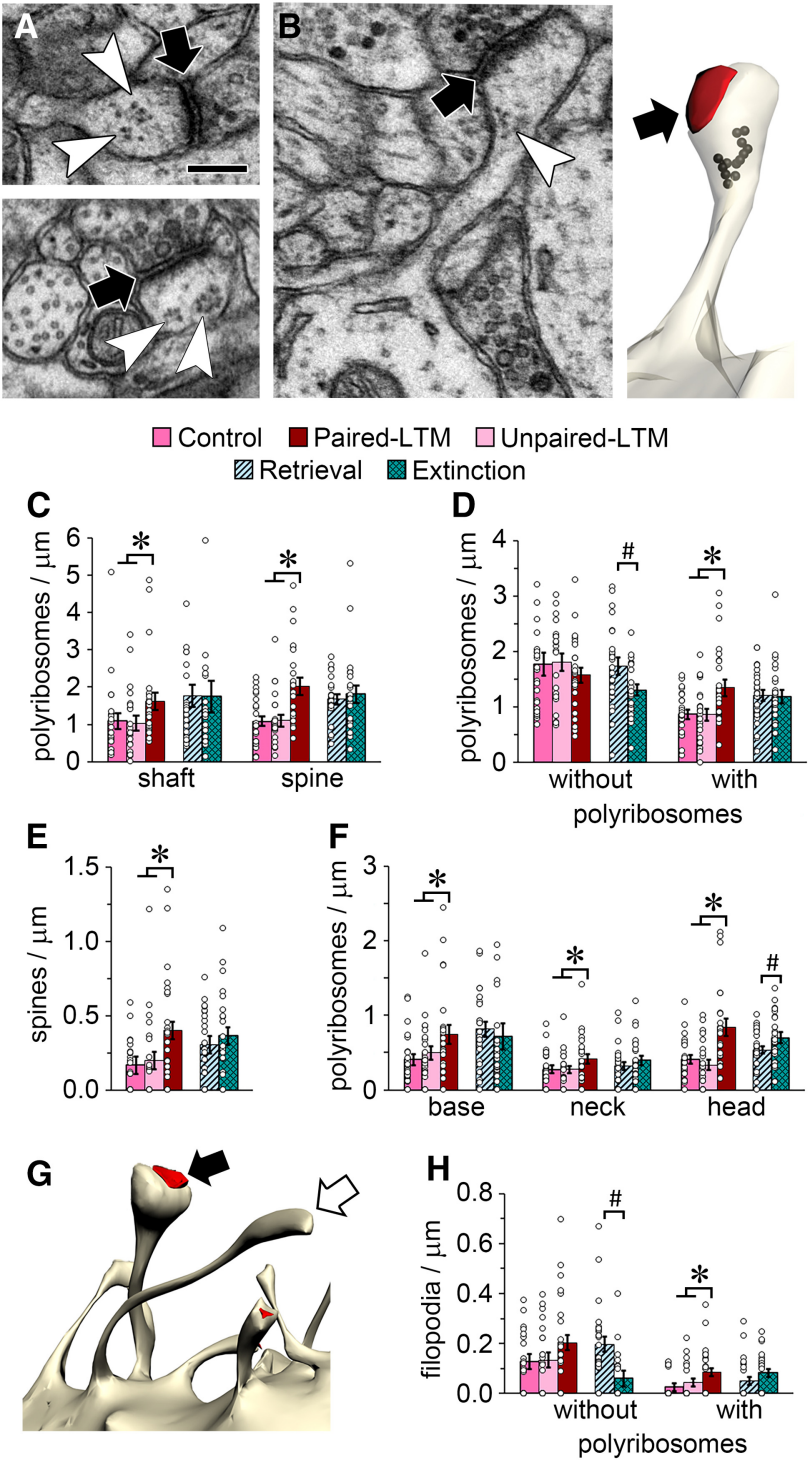
Up-regulation of dendritic polyribosomes during long-term memory. (*A*) Examples of polyribosomes (white arrowheads) in dendritic spines forming asymmetric synapses (black arrows). Scale bar, 200 nm. (*B*) A spine with a polyribosome in its head (*left*) and a reconstruction of the same spine (*right*) showing the location of the ribosomes (black) near the synapse (arrow). (*C*) There were more polyribosomes in dendritic spines and shafts in the paired-LTM group. (*D*) There were fewer spines without polyribosomes in the extinction group than the retrieval group, and more spines with polyribosomes in the paired-LTM group. (*E*) There were more spines with more than one polyribosome in the paired-LTM group. (*F*) There were more polyribosomes in spine bases, necks, and heads in the paired-LTM group, and more in spine heads in the extinction group relative to retrieval. (*G*) Reconstruction of a filopodium (white arrow) next to a spine with a synapse (black arrow). (*H*) There were fewer filopodia without polyribosomes in the extinction group versus the retrieval group, and more filopodia with polyribosomes in the paired-LTM group. (*) *P* < 0.05 for paired-LTM versus control and unpaired-LTM, (#) *P* < 0.05 for extinction versus retrieval.

Polyribosomes are sites of protein synthesis that appear distinctly in the EM as aggregates of ribosomes on an RNA strand (Slayter et al. 1963; Warner et al. 1963) and are readily observable in dendritic spines (Fig. 2A; Steward and Levy 1982). We used a stringent definition of polyribosomes as clusters of at least three ribosomes (Ostroff et al. 2002, 2010). Polyribosomes within the reconstructed dendrites (Fig. 2B) were quantified to determine whether the increases in polyribosomes observed shortly after paired training (Ostroff et al. 2010, 2017) would subside during the LTM phase. To the contrary, we found significantly more polyribosomes in dendritic shafts and spines of paired-LTM rats compared with unpaired-LTM and control rats, and no difference between the retrieval and extinction groups (Fig. 2C). There were no statistically significant effects of training on overall spine frequency (*P* = 0.34 and *P* = 0.12 for LTM and extinction, respectively) (data not shown), but effects emerged when spines with and without polyribosomes were analyzed separately (Fig. 2D). There were fewer spines without polyribosomes in the extinction group relative to the retrieval group, and more spines with polyribosomes in the paired-LTM group (Fig. 2D). In general, there were more polyribosomes in spines than spines with polyribosomes (Fig. 2C,D), reflecting the incidence of spines with multiple polyribosomes, and there were more of these spines in the paired-LTM group (Fig. 2E). Within dendritic spines, polyribosomes were found in the base (within 150 nm of the spine origin), neck, or head. Polyribosome frequency was higher in all three locations in the paired-LTM group, and in the head location in the extinction group relative to the retrieval group (Fig. 2F). In the overall data set ∼7% of dendritic protrusions lacked a synapse and were classified as filopodia (Fig. 2G). There were more filopodia with polyribosomes in the paired-LTM group and fewer without polyribosomes in the extinction group relative to the retrieval group (Fig. 2H).

### Learning effects differ between spines of different sizes

**Figure 3.**
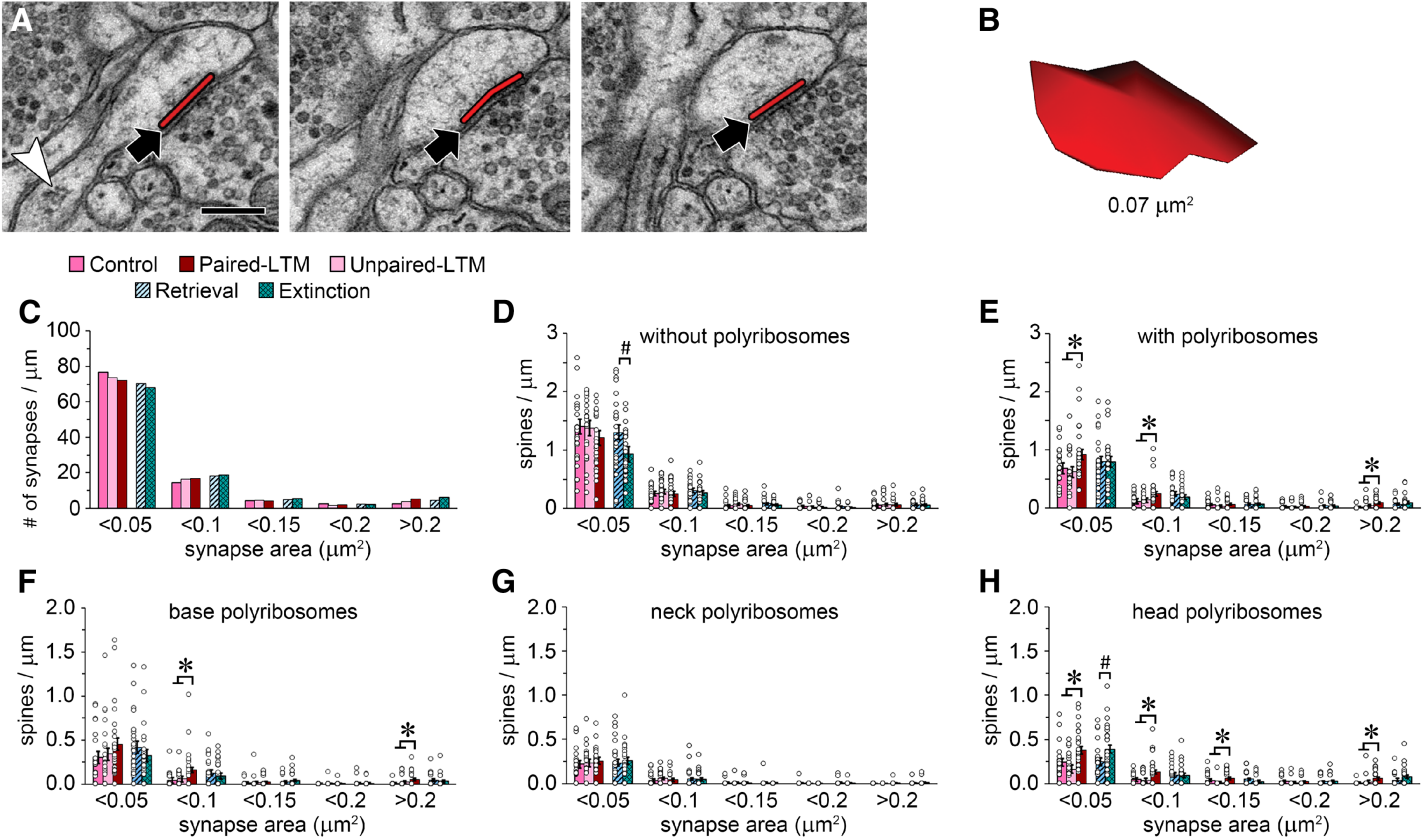
Effects of training on synapse size. (*A*) Three consecutive serial sections of a synapse (arrows) on a dendritic spine, with a line marking the length of the postsynaptic density (PSD). A polyribosome (arrowhead) is visible in the spine's neck in the *left* panel. Scale bar, 250 nm. (*B*) Reconstruction of the PSD in *A*, which has an area of 0.07 µm^2^. (*C*) Histogram of synapse size in all training groups. (*D*) Among spines without polyribosomes, the extinction group had fewer with PSD areas <0.05 µm^2^ relative to the retrieval group. (*E*) Among spines with polyribosomes, there were more spines in the paired-LTM group with PSD areas <0.05 µm^2^, 0.05–0.1 µm^2^, and >0.2 µm^2^. (*F*) In the paired-LTM group, there were more spines with base polyribosomes and PSD area of 0.05–0.1 µm^2^ and >0.2 µm^2^. (*G*) There were no group differences among spines with neck polyribosomes. (*H*) In the extinction group, there were more spines with head polyribosomes and PSD area <0.05 µm^2^ relative to the retrieval group. In the paired-LTM group, there were more spines with head polyribosomes and PSD area <0.05 µm^2^, 0.05–0.1 µm^2^, 0.1–0.15 µm^2^, and >0.2 µm^2^. (*) *P* < 0.05 for paired-LTM versus control and unpaired-LTM, (#) *P* < 0.05 for extinction versus retrieval.

Synapse size, spine size, and synapse strength are all positively correlated, and enlarged spines are considered likely sites of memory storage (Harris and Stevens 1989; Bourne and Harris 2007; Kasai and Fukuda 2010; Ostroff et al. 2010; Segal 2017). Synapse size was assessed by measuring each postsynaptic density (PSD) and calculating its two-dimensional area across serial sections (Fig. 3A,B). Because synapse size is not normally distributed (Fig. 3C) and spines on either end of the distribution may have different functions, overall means do not provide a meaningful picture of the spine population. Spines were therefore binned by synapse size for frequency comparisons. For spines without polyribosomes, the lower frequency in the extinction relative to the retrieval group (Fig. 2F) turned out to be specific to the smallest spines (Fig. 3D). For spines with polyribosomes, the higher frequency in the paired-LTM group (Fig. 2F) was seen in the largest and in the two smallest size bins (Fig. 3E). To assess effects on polyribosome localization across the size distribution, spine frequency was compared for each polyribosome location separately. For this analysis, each spine was counted once for every location where it contained at least one polyribosome. There was an effect of LTM on spines with polyribosomes at the base in the largest and second-smallest size bins (Fig. 3F). There were no training effects on the frequency of spines with polyribosomes in the neck (Fig. 3G), indicating that the LTM effect on polyribosomes in this location (Fig. 2H) was solely due to an increase in spines with multiple neck polyribosomes. For spines with polyribosomes in the head, there was an LTM effect in four of the five size bins, but the retrieval and extinction groups differed only in the smallest (Fig. 3H). Overall, there were effects of paired training across the entire range of synapse sizes, while only the smallest spines differed between retrieval and extinction.

### Effects of paired training are independent of other spine features

**Figure 4.**
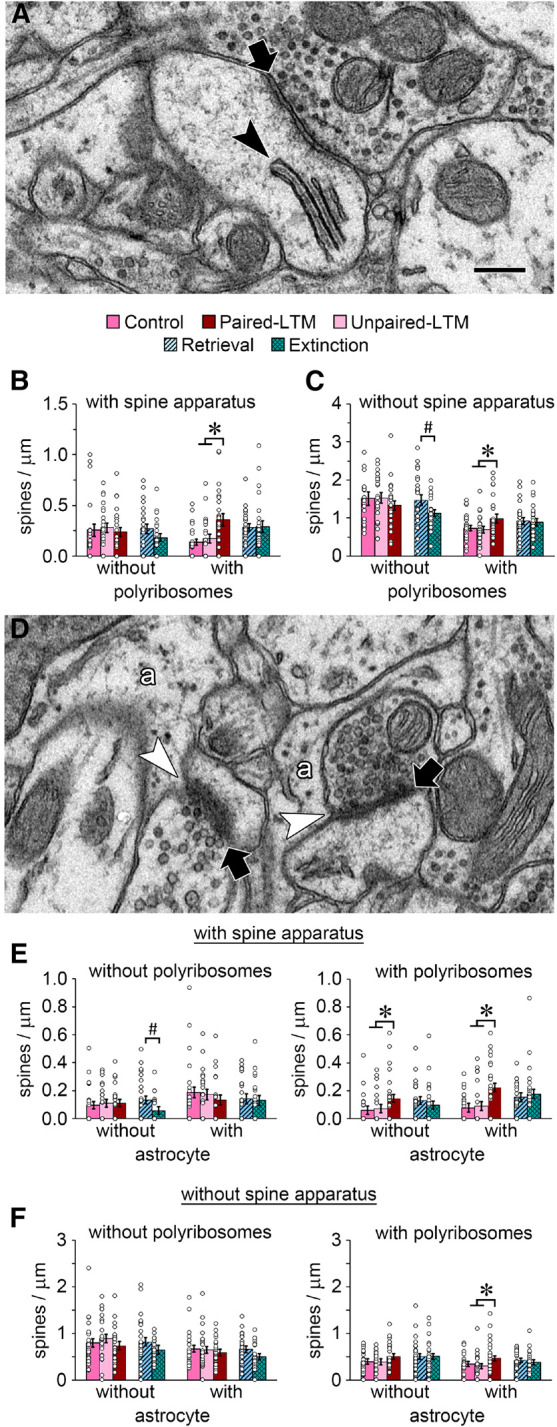
Spine apparatus and astrocytes at synapses. (*A*) EM of a spine apparatus (black arrowhead) in a spine forming an asymmetric synapse (arrow). (*B*) There were more spines with a spine apparatus and polyribosomes in the paired-LTM group. (*C*) Among spines without a spine apparatus, there were fewer spines without polyribosomes in the extinction group than in the retrieval group, and more spines with polyribosomes in the paired-LTM group. (*D*) EM showing two spine heads with asymmetric synapses (arrows) with astrocytic processes (a) making direct contact (white arrowheads) with the synaptic cleft. The spine head at the *right* contains a spine apparatus. (*E*) For spines with a spine apparatus, there were fewer without polyribosomes or astrocytes in the extinction group relative to the retrieval group, and more with polyribosomes both with and without astrocytes in the paired-LTM group. (*F*) For spines without a spine apparatus, there were more with both polyribosomes and astrocytes in the paired-LTM group. (*) *P* < 0.05 for paired-LTM versus control and unpaired-LTM, (#) *P* < 0.05 for extinction versus retrieval. Scale bar in *A* and *D*, 250 nm.

The spine apparatus (Fig. 4A) is a specialization of the smooth endoplasmic reticulum that is composed of calcium-containing cisterns interleaved with dense actin plates (Fifkova et al. 1983; Spacek 1985; Deller et al. 2000; Capani et al. 2001). The spine apparatus is associated with large, mature spines (Spacek and Harris 1997; Ostroff et al. 2012) and although its exact function is unknown, it may be involved in spine stabilization and long-term memory (Deller et al. 2007). We have previously reported an increased frequency of polyribosomes in spines with and without a spine apparatus during consolidation of auditory Pavlovian conditioning (Ostroff et al. 2010, 2017). We hypothesized that during the long-term memory phase, polyribosomes would be preferentially associated with larger, presumably stable spines versus smaller spines, but this was not the case (Fig. 3D). In the LA, the spine apparatus is always present in the largest spines (PSD area >0.1 µm^2^) but appears in some small spines as well (Gindina et al. 2021). Thus, we wondered whether the spine apparatus would account for the extra spines with polyribosomes, regardless of size. There were indeed more spines with both polyribosomes and a spine apparatus in the paired-LTM group (Fig. 4B), as expected from the effect in the largest spines (Fig. 3E), but there were also more spines with polyribosomes and no spine apparatus (Fig. 4C). In contrast, the decrease in small spines without polyribosomes in the extinction group relative to the retrieval group (Fig. 3D) was specific to spines without a spine apparatus (Fig. 4C).

Another variable aspect of synapse structure that may be associated with plasticity and stability is the involvement of astrocytic processes, which are present throughout the neuropil and sometimes make direct contact with the synaptic cleft (Fig. 4D). EM studies have found that these contacts occur in roughly half of asymmetric synapses in the LA and hippocampus and are more common in large spines (Ventura and Harris 1999; Witcher et al. 2010; Ostroff et al. 2014). In an earlier study, we found that spine proliferation during consolidation of Pavlovian conditioning was specific to spines without astrocytic contact at the synapse (Ostroff et al. 2014), suggesting that astrocytes are associated with stable synapses as opposed to those undergoing active plasticity processes. If this is the case, astrocytic contacts might be more prevalent, rather than less, during the long-term memory phase. To examine the distribution of astrocytic contacts, the perimeter of each synapse was examined for the presence or absence of an astrocytic process at the synaptic cleft. When the presence of the spine apparatus and polyribosomes was taken into account, there were fewer spines with a spine apparatus but no polyribosomes or astrocytes in the extinction group versus the retrieval group (Fig. 4E). In contrast, the greater number of spines with a spine apparatus and polyribosomes in the paired-LTM group (Fig. 4B) was present in spines with and without astrocytic contacts (Fig. 4E). For spines without a spine apparatus, the group differences (Fig. 4C) were reflected in the means regardless of astrocyte contact, although the only statistically significant effect was a greater number of spines with both polyribosomes and astrocytes (Fig. 4F). As was the case with synapse size, grouping spines by their morphological features revealed no specificity in the effects of paired training, while only select spines differed between extinction and retrieval.

### Fewer small shaft synapses after extinction versus retrieval

**Figure 5.**
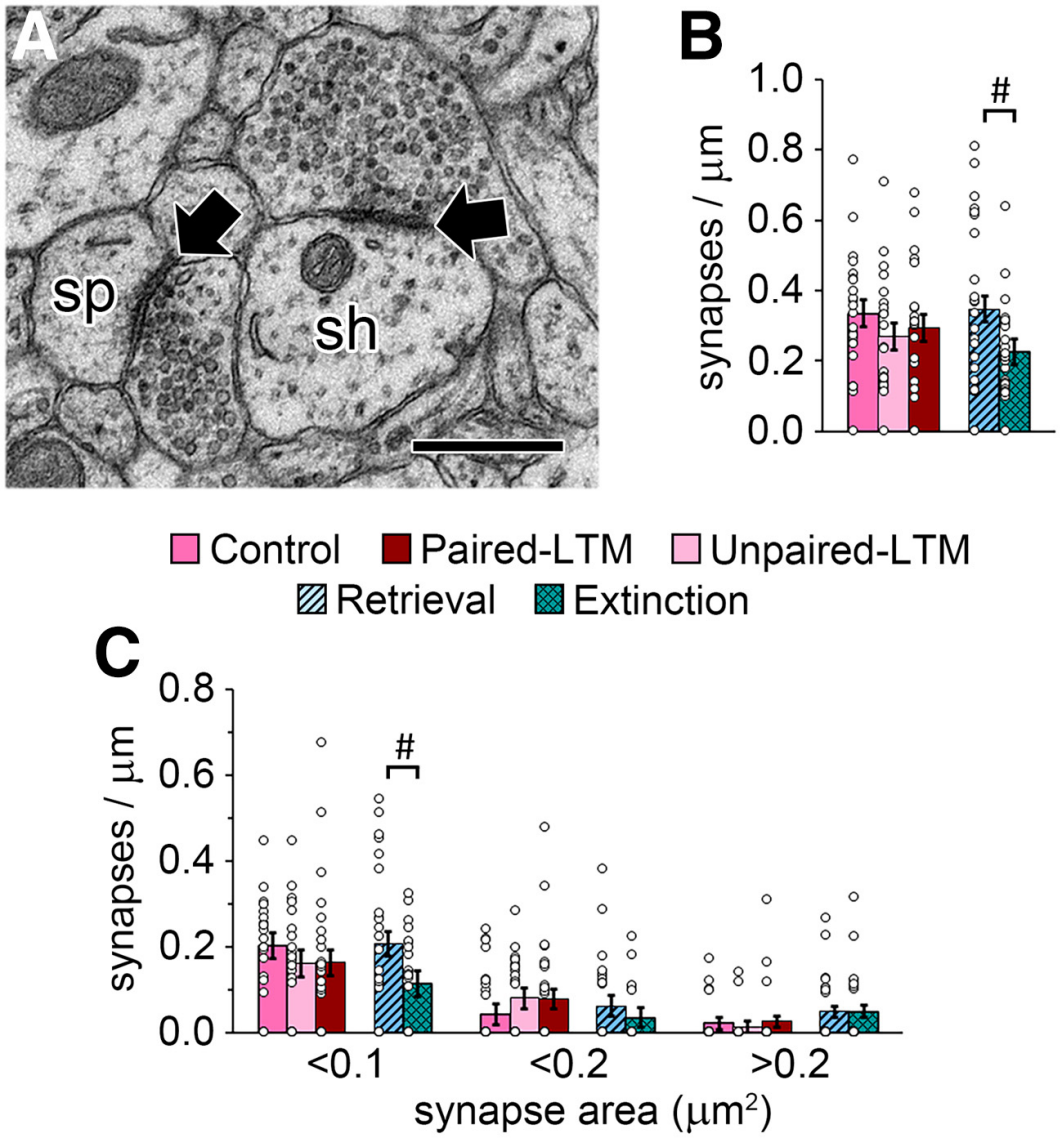
Shaft synapses and total synapse area. (*A*) EM image of asymmetric synapses (arrows) on a dendritic shaft (sh) and a spine (sp). (*B*) There were fewer shaft synapses in the extinction group relative to the retrieval group. (*C*) Shaft synapse frequency binned by synapse area, showing an effect of extinction versus retrieval in the smallest bin. (#) *P* < 0.05.

Approximately 9% of asymmetric synapses on the dendrites in our data set were on dendritic shafts as opposed to protrusions (Fig. 5A). In a previous study of LA dendrites, we found that all axons that form asymmetric shaft synapses also form spine synapses (Ostroff et al. 2012), so although we did not reconstruct these axons, they are likely from the same population as the spine synapses that we analyzed. There was no effect of paired training on the frequency of asymmetric shaft synapses, but there were fewer in the extinction group relative to the retrieval group (Fig. 5B). When these synapses were binned by size, the effect was present only for the smallest synapses (Fig. 5C). Thus, the extinction group had fewer small synapses than the retrieval group in every context that we examined.

### Correlates of shaft polyribosomes

**Figure 6.**
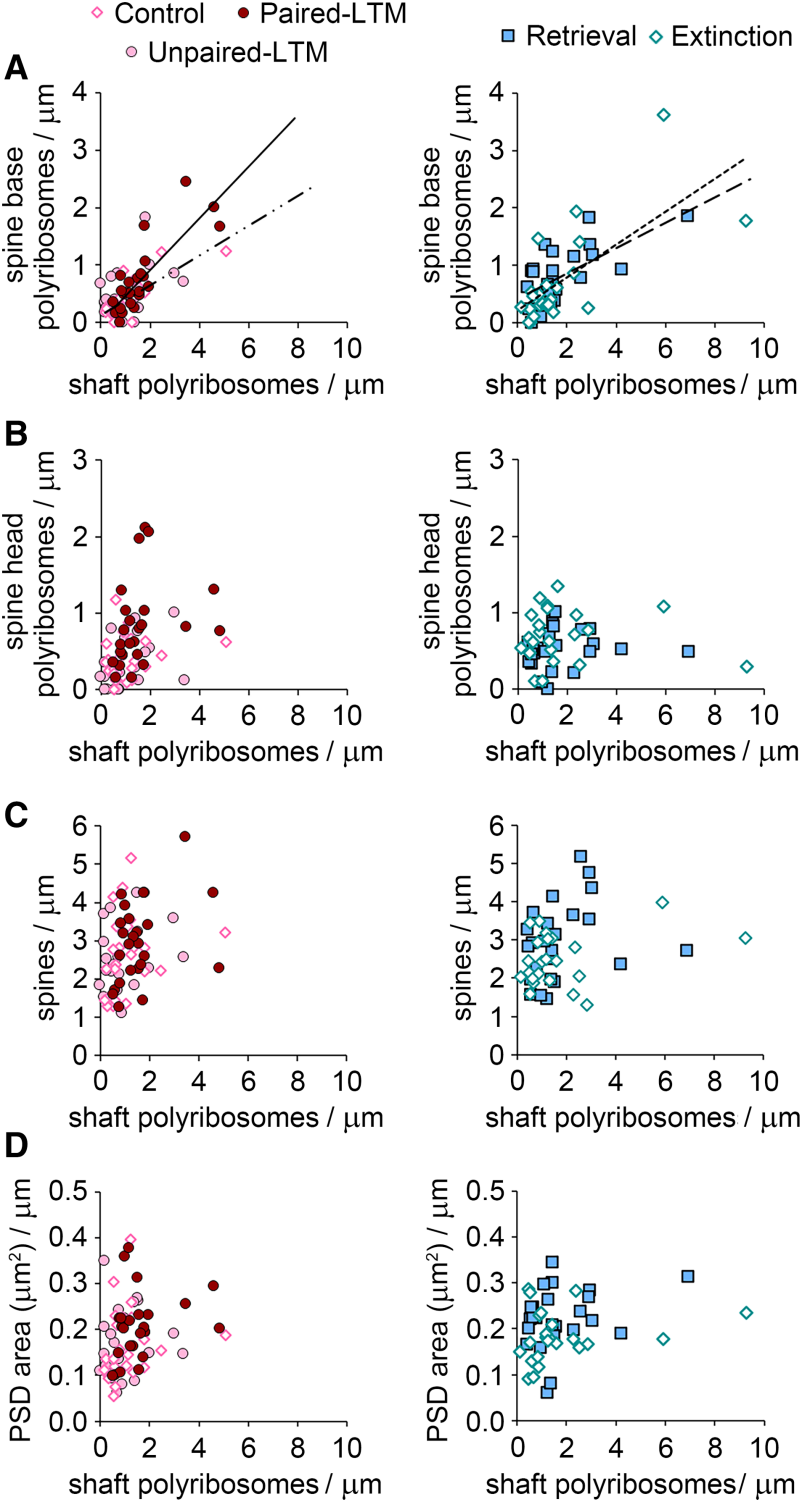
Correlates of shaft polyribosome frequency. (*A*) Shaft polyribosomes correlated with polyribosomes in the spine base in the control (dashed line: *r*^2^ = 0.60, *P* = 0.00002), paired-LTM (solid line: *r*^2^ = 0.69, *P* = 0.00001), retrieval (dashed line: *r*^2^ = 0.42, *P* = 0.0006), and extinction (dotted line: *r*^2^ = 0.49, *P* = 0.0002) groups. (*B*) Shaft polyribosomes and spine head polyribosomes were uncorrelated in all groups. (*C*) Shaft polyribosomes were uncorrelated with overall spine frequency in all groups. (*D*) Shaft polyribosomes were uncorrelated with total PSD area per micrometer of dendrite in all groups.

Polyribosomes occur with similar frequency in dendritic shafts and spines (Fig. 2E), but whether these represent separate processes or a single pool that is regulated collectively is an open question. The presence of translation initiation factors in dendritic spine heads (Gindina et al. 2021) suggests that some translational control is compartmentalized at the level of individual synapses. On the other hand, synapses in the same compartment can share new proteins during plasticity (Sajikumar et al. 2007), which could reflect a common pool of polyribosomes. Consistent with the common pool model, we found in an earlier study that the frequency of polyribosomes in LA dendritic shafts and spines was positively correlated regardless of training (Ostroff et al. 2010). The same correlation was observed in each of the groups in the present study (*r*^2^ = 0.30, *P* = 0.000001 for all groups pooled) (data not shown). Because the spine head location is more unambiguously associated with individual synapses and may be capable of independent regulation, we examined the spine base and head separately. Shaft polyribosomes were correlated with spine base polyribosomes in all groups except the unpaired-LTM group (Fig. 6A), but never with spine head polyribosomes (Fig. 6B), and there were no correlations between spine head and spine base polyribosomes (data not shown). The correlation between shaft and spine base polyribosomes could be an artifact of where the boundary between the base and shaft was set, but this seems unlikely given the stringent cutoff of 150 nm. Shaft polyribosomes could reflect the need for proteins to support spine maintenance or learning-related spinogenesis, in which case their frequency might be expected to scale with spine numbers. There were no correlations, however, between shaft polyribosomes and spine frequency (Fig. 6C) or total PSD area per length of dendrite (Fig. 6D). Overall, these data suggest that shaft polyribosomes are regulated independently of those in spine heads and are not related to spine and synapse numbers.

**Figure 7.**
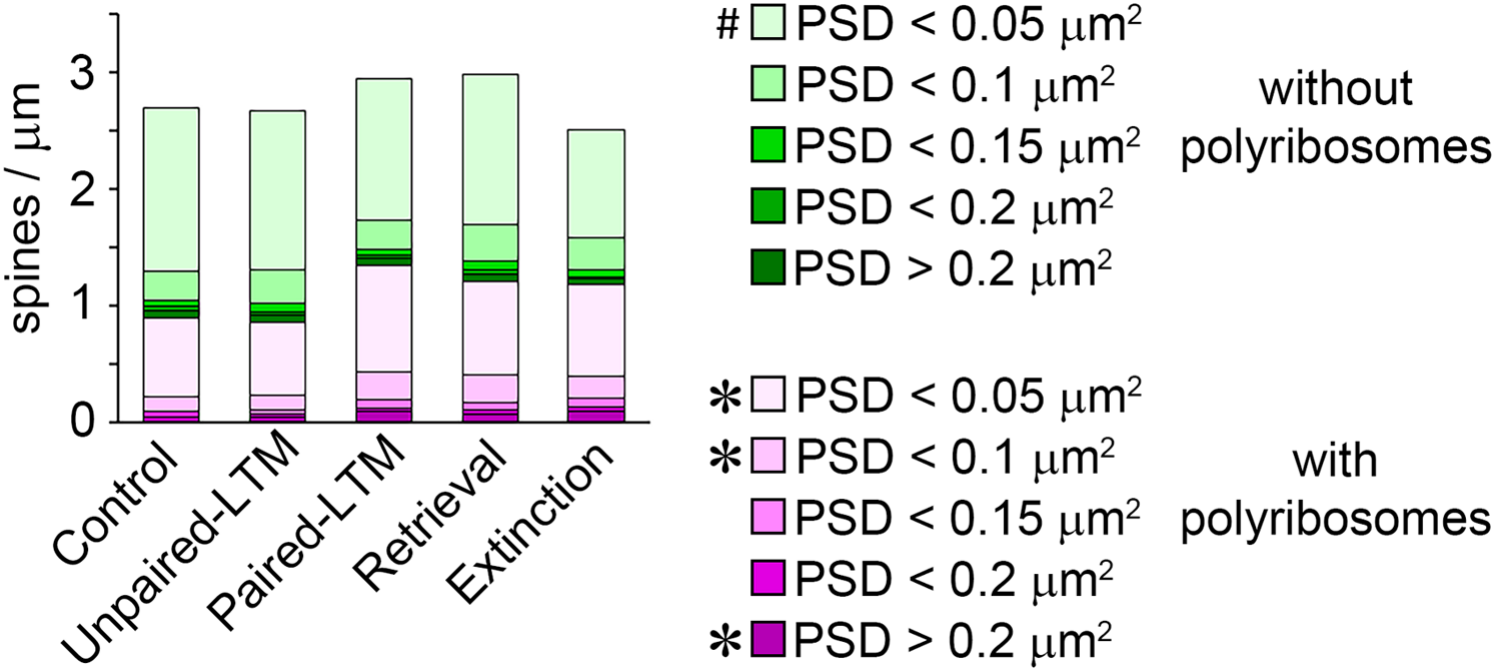
Summary of spine frequency by PSD area and polyribosomes. (*) *P* < 0.05 for paired-LTM versus control and unpaired-LTM, (#) *P* < 0.05 for extinction versus retrieval.

The mean spine frequencies by synapse size and polyribosome content for each of the five experimental groups are shown in Figure 7. The overall effect of paired training on spine frequency was an up-regulation of polyribosome-containing spines of all sizes, whereas the only difference between retrieval and extinction was that the extinction group had fewer very small spines that lacked polyribosomes. Initial conditioning and extinction training thus have nearly orthogonal effects on the spine population.

## Discussion

Memory consolidation requires protein synthesis within a short time window after learning (Davis and Squire 1984; Rosenberg et al. 2014). Pavlovian conditioning memory is impaired by protein synthesis inhibitors infused into the LA immediately after learning, but not 6 or 24 h later (Nader et al. 2000; Schafe and LeDoux 2000; Maren et al. 2003), and the same time course is seen in the hippocampus after contextual conditioning (Bourtchouladze et al. 1998). New proteins synthesized in the hippocampus after avoidance learning are only available to stabilize new memories for ∼1 h (Moncada et al. 2015), indicating that both the production and functional incorporation of new proteins are restricted to the early posttraining period. The short time window is suggestive of local translation, which can provide proteins rapidly at sites far from the nucleus and is known to be important for synaptic plasticity and memory (Holt and Schuman 2013). In previous work, we observed up-regulation of polyribosomes in LA dendrites 1 h after Pavlovian conditioning (Ostroff et al. 2010, 2017), consistent with a local source of proteins to support consolidation-related synaptic changes. If this is the case, the extra polyribosomes should not persist into the translation-independent long-term memory phase. Contrary to that prediction, we found that polyribosomes were elevated in LA dendrites 24 h after Pavlovian conditioning.

Memories are considered stable after 24 h based on their resistance to single infusions of protein synthesis inhibitors, meaning that a short-lived reduction in translation does not permanently disrupt memory. Inhibiting translation right after learning derails consolidation-specific gene expression cascades that are dependent on transiently activated signaling pathways (Kandel et al. 2014; Rosenberg et al. 2014). Past this point, synapses may still need an ongoing supply of proteins to maintain learning-induced changes, but the rate of protein turnover may not be high enough to reverse synaptic changes before the drug wears off. Levels of a number of synaptic proteins have been found to be altered in the LA 24 h after Pavlovian conditioning (Hong et al. 2013), which is consistent with a role for ongoing translational changes. Since large synapses are stronger and thus could represent learning-related potentiation (Nusser 2000; Matsuzaki et al. 2004; Bourne and Harris 2007; Nicholson and Geinisman 2009), we hypothesized that if polyribosomes remained up-regulated in the long-term memory phase they would be associated with the largest spines. Likewise, because the spine apparatus is associated with learning and memory (Deller et al. 2007) and is present in all large spines (Gindina et al. 2021), we hypothesized that excess polyribosomes would be associated with the spine apparatus. Contrary to these expectations, polyribosomes remained up-regulated in spines of all sizes and in spines with and without a spine apparatus. This suggests that spine polyribosomes do not simply represent an ongoing need for extra proteins at learning-potentiated synapses.

Although polyribosomes have long been recognized as sites of protein synthesis (Slayter et al. 1963; Warner et al. 1963), there is mounting evidence that (1) not all polyribosomes are actively translating, and (2) not all active translation occurs on polyribosomes. Once loaded onto an mRNA strand, ribosomes can be stalled in place for later reactivation (Richter and Coller 2015), and a study of cultured neurons found that unlike polyribosomes in cell bodies, the majority of polyribosomes in dendrites were stalled (Langille et al. 2019). It is possible that learning not only triggers distribution of dormant polyribosomes to sites that need proteins immediately but also stations them at sites likely to need them in response to future events. This could explain why we found more polyribosomes not only in large spines and spines with a spine apparatus—presumably the ones that represent memory-related potentiation—but also in dendritic shafts and small spines, which could be primed for future plasticity. It has also emerged recently that translation frequently occurs on monosomes, and that this is especially true in dendrites and is selective for certain mRNAs (Heyer and Moore 2016; Biever et al. 2020). The sustained presence of polyribosomes could thus also signal a rapid and persistent shift toward polyribosome-associated transcripts. Monosomes are not readily detectable by their EM morphology, so if the predicted pattern of transient up-regulation of translation is carried by monosomes, we would not have detected it.

Posttraining inhibition of cap-dependent translation initiation, the process by which most ribosomes are loaded onto mRNA, impairs consolidation of Pavlovian conditioning and associated up-regulation of polyribosomes in spine heads and dendritic shafts 1 h after training (Hoeffer et al. 2011; Ostroff et al. 2017). A possible scenario is that newly assembled polyribosomes accumulate in dendrites during consolidation and remain there in a dormant state, perhaps allowing a different complement of proteins to be delivered during future plasticity events. Postretrieval inhibition of overall protein synthesis in the LA impairs reconsolidation (Nader et al. 2000; Duvarci et al. 2005; Doyere et al. 2007), but one study found that inhibition of the predominant initiation mechanism does not (Hoeffer et al. 2011). This could mean that translation of pre-existing polyribosomes supports reconsolidation, and we hypothesized that if this is the case, retrieval would deplete dendritic polyribosomes. Although we did not expose our long-term memory subjects to the retrieval chamber and thus did not directly compare them with the retrieval subjects, it is notable that mean polyribosome frequency was similar between the two groups except for a lower frequency of polyribosomes in small spine heads in the retrieval group. If activation of synapses during retrieval depletes polyribosomes, this should have been more evident in the extinction group, which received 48 tones instead of the two tones presented to the retrieval group. Polyribosome frequency did not differ between the two groups, however, except for a higher frequency of polyribosomes in small spine heads in the extinction group. Overall, dendritic polyribosomes appeared surprisingly stable after initial consolidation.

We examined excitatory synapses on the dendrites of excitatory neurons in the dorsal LA, which are known to exhibit LTP-like plasticity after Pavlovian conditioning and depotentiate when reconsolidation is blocked (Rogan et al. 1997, 2005; Doyere et al. 2007; Sigurdsson et al. 2007). It is unclear whether extinction learning involves depotentiation of the same inputs, recruitment of heterosynaptic inhibition, or both. Recovery phenomena like renewal, reinstatement, and spontaneous recovery demonstrate that the original memory remains in some form (Bouton 2002; Myers and Davis 2002). In favor of an “erasure” model, there is evidence for extinction-induced depotentiation of inputs to dorsal LA weakening the engram (Lin et al. 2003a,b; Kim et al. 2007; Hong et al. 2009). In this model, metaplasticity allows for rapid repotentiation of LA inputs to account for recovery phenomena (Lee et al. 2013). Alternative models hypothesize that extinction spares the original memory trace, and strengthening of amygdala inhibitory circuits mediates response suppression (for review, see Maren 2015). Recovery phenomena are explained by context-dependent gating of new inhibitory learning, usually via effects of extra-amygdala inputs to inhibitory intercalated cells positioned between the LA and central amygdala (Sotres-Bayon and Quirk 2010; Duvarci and Pare 2014). Postextinction inhibition of protein synthesis in the LA impairs reconsolidation, making it difficult to assess effects on extinction consolidation (Duvarci et al. 2006). Pre-extinction inhibition of protein synthesis with systemic anisomycin impairs extinction consolidation but not reconsolidation (Suzuki et al. 2004), though this could be affecting other extinction-relevant brain regions like the infralimbic cortex (Milad and Quirk 2012). Extinction has also been reported to halt (Suzuki et al. 2004) or impair (Monfils et al. 2009) reconsolidation, depending on the protocol. Thus, it is difficult to predict how extinction might change polyribosomes in dendrites of LA cells that appear to remain elevated 24 h after conditioning.

Relative to just retrieval, extinction resulted in a loss of dendritic spines, consistent with erasure. However, this was specific to small spines that did not contain polyribosomes. Since large spines were preserved, our data may indicate turnover of small spines or diversion of resources to existing synaptic connections, both of which would be consistent with metaplastic changes but not with dismantling of a memory trace. In a previous study, we found a reduction in LA synapse size 1 h after conditioned inhibition training (Ostroff et al. 2010), consistent with the reduction in synaptic responses in this paradigm (Rogan et al. 1997, 2005). Extinction-related depotentiation occurs much sooner than our 1-h posttraining time point (Kim et al. 2007; Hong et al. 2009), and we would expect to see a similar decrease in synapse size if erasure is indeed occurring. The increased number of polyribosomes in small spine heads after extinction parallels our previous observations at the same time point after initial training (Ostroff et al. 2010, 2017). This could suggest early stages of synapse strengthening after extinction, but could also mean that polyribosomes are delivered to synapses under specific conditions like the contingency violations in both conditioning and extinction. Overall, our results demonstrate that dendritic polyribosomes remain up-regulated into the long-term memory phase and are largely unaffected by activation of a memory. Instead of active translation, these polyribosomes may represent metaplastic changes in translation capacity in the vicinity of potentiated synapses. Our data further support retention of an initial memory trace after extinction training, as we found no loss of large synapses.

It is important to keep in mind limitations of the present analyses when considering implications for learning, consolidation, retrieval, reconsolidation, and extinction of threat conditioning. First, because the ssTEM method limits the number of samples that can be collected in a single experiment, we did not include a nonretrieval LTM control group with exposure to the retrieval chamber, which prevented direct comparison between a retrieved and a nonretrieved memory. In addition, these data reflect a snapshot in time focused on excitatory neuron dendrites in a small region of the LA, whereas Pavlovian conditioning involves distributed plasticity in multiple brain regions. This is also true of extinction, where consolidation and retrieval depend critically on the infralimbic cortex and hippocampus (Milad and Quirk 2002; Marek et al. 2018). Plasticity of inhibitory transmission in the BLA and adjacent intercalated cells is also known to play a key role in extinction (Maren 2015; Bouton et al. 2021). Ongoing analyses of local inhibitory transmission in the same LA region may shed more light on these processes, and future studies may examine related changes in other brain regions.

## Materials and Methods

### Subjects and behavior

Subjects were adult male Sprague-Dawley rats (Hilltop Lab Animals, Inc.) weighing ∼300 g, housed singly on a 12-h light/dark cycle with ad libitum food and water. All procedures were approved by the New York University Animal Care and Use Committee. Experiments were conducted during the animals’ light cycle, and all animals spent exactly 1 wk in the vivarium between arrival from the vendor and the start of the experiment. Two batches of animals were trained 1 wk apart, with each batch representing half of each of the five experimental groups. The conditioning chambers and Pavlovian conditioning protocols were as previously described (Ostroff et al. 2010). All rats were habituated to square conditioning chambers (Coulbourn Instruments; context A) for 30 min on two consecutive days, and then randomized into one of five training groups: control (*n* = 4), unpaired-LTM (*n* = 6), paired-LTM (*n* = 6), retrieval (*n* = 8), and extinction (*n* = 16). Paired training consisted of five 30-sec, 5-kHz, 80-dB tones coterminating in a 1-sec, 0.7-mA scrambled footshock (5-min mean intertrial interval), and unpaired training consisted of five nonoverlapping tones and footshocks (119-sec mean shock-to-tone interval; 180-sec mean tone-to-shock interval). Extinction and retrieval were conducted in novel rectangular test chambers (Med Associates; context B). Extinction consisted of 48 tones at a 5-sec interval, and retrieval consisted of two tones corresponding to the times of the first and last tones of extinction. Temporally massed trials were chosen to maximize extinction learning during a single, relatively short session (Cain et al. 2003). Two tones were used for the retrieval group to control for time-dependent processes corresponding to first and last tone exposure using a protocol that does not induce extinction. All sessions lasted 32.5 min. For validation of the extinction protocol, a separate group of male rats (*n* = 16) received paired training, extinction training, and an extinction test (five tones using 5-min intervals) on three consecutive days using an ABB design. For all training and testing, freezing during each tone or the 30-sec pretone period was rated manually from video recordings. Because it is not practically feasible to include large numbers of animals in an ssTEM experiment, our tissue collection strategy was designed to minimize variability between subjects and avoid using behavioral outliers for analysis. Freezing during the training sessions was rated for each animal, and the four animals whose behavior was closest to the mean for each group were selected for EM processing. Of these four, the three with the highest-quality ultrastructural preservation were chosen for serial EM reconstruction.

### Serial section transmission electron microscopy

Tissue preparation for serial EM was identical to that used in Ostroff et al. (2010). Chemicals were obtained from Electron Microscopy Sciences unless otherwise stated. Animals were deeply anesthetized and perfused transcardially with mixed aldehydes, and the brains were sectioned at 70 µm on a vibrating slicer (Leica). Sections containing the lateral amygdala were postfixed in osmium, stained en bloc with uranyl acetate, and flat-embedded in LX-112 epon resin (Ladd Research Industries). Serial sections of uniform thickness were cut on an ultramicrotome (Leica), picked up on slot grids coated with pioloform (Ted Pella), and stained with aqueous uranyl acetate and Reynold's lead citrate. Sections were imaged at 7500× on a JEOL 1230 transmission electron microscope with a Gatan Ultrascan 4000 digital camera.

### Reconstruction and analysis

Reconstruct software (RRID:SCR_002716; Fiala 2005) was used for all digital image alignments, reconstructions, and measurements. One series per rat was cut and imaged, with an average of 159 ± 2 imaged sections (range 148–180) per series. Section thickness was estimated using mitochondrial diameters (Fiala and Harris 2001) and averaged 56 nm ± 1 nm (range 50–60). Dendritic segments that were in cross-section and whose protrusions were contained in the series were selected for reconstruction. Spiny dendrites presumably belonging to excitatory projection neurons (McDonald 1982, 1992) were used for analysis, while large apical dendrites and aspinous dendrites from putative inhibitory neurons (McDonald and Pearson 1989) were excluded. There were seven or eight dendrites analyzed in each series, for a total of 116 dendrites with 2918 synapses. Breakdowns of dendrite and synapse numbers by group and rat are in Table 1. For unbiased frequency measurements along dendrites, the ventral end of the series was designated as the inclusion end. Dendrites were analyzed between the first complete protrusion on the inclusion end and the first incomplete protrusion on the exclusion end with an average inclusion length of 8 µm ± 0.2 µm (range 3.9–11.9). There were no differences between experimental groups in series length, section thickness, or dendrite length. Presumed excitatory synapses were identified by standard criteria, including asymmetric morphology and round synaptic vesicles (Gray 1959). Dendritic protrusions bearing at least one asymmetric synapse were defined as spines, and nonsynaptic protrusions were defined as filopodia. Approximately 10% of protrusion origins gave rise to more than one protrusion, and 4% of spines carried more than one synapse. There were no group differences in the incidence of these types and they were not examined separately.

**Table 1.**
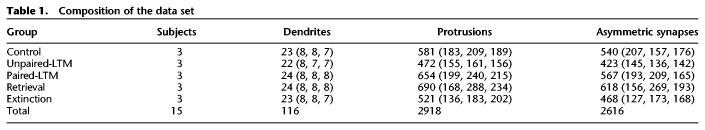
Composition of the data set

### Statistics

For ssTEM measurements, group means were compared using hierarchical ANOVAs with subject nested into group to account for intersubject variability. Because the control, unpaired-LTM, and paired-LTM groups were not handled on the second day of the experiment, they were compared with only each other, and the retrieval and extinction groups were separately compared with each other. There were no differences between the control and unpaired-LTM groups in any of the measures, so these groups were pooled for comparison with the paired-LTM group. In the text, comparisons of the paired-LTM group with unpaired-LTM and control are referred to as LTM effects, and comparisons of the retrieval and extinction groups are referred to as extinction effects. Exact values for *F*, *P*, and partial η^2^ are in Supplemental Table S1. For the extinction LTM test, means were compared using a paired two-tailed *t*-test and results are in the legend for Figure 1. Results of simple linear regression analyses are in the legend for Figure 6.

### Competing interest statement

The authors declare no competing interests.

## Supplementary Material

Supplemental Material
