# Supplementary material for: Persistent up-regulation of polyribosomes at synapses during long-term memory, reconsolidation, and extinction of associative memory
Source: Learn Mem. 2022 Aug;29(8):192–202. doi: 10.1101/lm.053577.122 (PMC9374273; doi:10.1101/lm.053577.122)
Supplement: Supplemental Material [file supp_29_8_192__DC1.html]

Supplemental Material 

# Persistent up-regulation of polyribosomes at synapses during long-term memory, reconsolidation, and extinction of associative memory

## Supplemental Material

- Supplemental\_Material.docx
