# Supplementary material for: Persistent up-regulation of polyribosomes at synapses during long-term memory, reconsolidation, and extinction of associative memory
Source: Learn Mem. 2022 Aug;29(8):192–202. doi: 10.1101/lm.053577.122 (PMC9374273; doi:10.1101/lm.053577.122)
Supplement: Supplemental Material [file supp_29.8.192_Supplemental_Material.docx]

| **Supplemental Table S1. Effects of Training** | | | | | | |  |  |  |  |  |  |  |  |
| --- | --- | --- | --- | --- | --- | --- | --- | --- | --- | --- | --- | --- | --- | --- |
| **Figure** | **Variable**  **(per μm)** | **LTM effect** | | | **subject (LTM) effect** | | | **extinction effect** | | | **subject (extinction) effect** | | |  |
|  |  | **F** | **p** | **partial η^2^** | **F** | **p** | **partial η^2^** | **F** | **p** | **partial η^2^** | **F** | **p** | **partial η^2^** | |
| 2C | shaft polyribosomes | 4.87 | 0.0312 | 0.075 | 1.28 | 0.2745 | 0.130 | 0.00 | 0.9472 | 0.000 | 0.28 | 0.8888 | 0.027 | |
| 2C | spine polyribosomes | 18.43 | 0.0001 | 0.235 | 1.04 | 0.4124 | 0.108 | 0.25 | 0.6165 | 0.006 | 0.69 | 0.5999 | 0.063 | |
| 2D | spines with polyribosomes | 11.32 | 0.0013 | 0.159 | 1.09 | 0.3840 | 0.112 | 0.02 | 0.8757 | 0.001 | 1.39 | 0.2538 | 0.120 | |
| 2D | spines without polyribosomes | 1.28 | 0.2623 | 0.021 | 2.11 | 0.0562 | 0.197 | 6.14 | 0.0174 | 0.130 | 2.18 | 0.0882 | 0.175 | |
| 2E | spines >1 polyribosome | 11.98 | 0.0010 | 0.169 | 1.99 | 0.0721 | 0.191 | 0.30 | 0.5896 | 0.007 | 1.28 | 0.2920 | 0.111 | |
| 2F | spine base polyribosomes | 6.17 | 0.0158 | 0.093 | 0.99 | 0.4470 | 0.104 | 0.29 | 0.5919 | 0.007 | 1.18 | 0.3357 | 0.103 | |
| 2F | spine neck polyribosomes | 4.30 | 0.0423 | 0.067 | 0.81 | 0.5836 | 0.086 | 0.75 | 0.3925 | 0.018 | 0.62 | 0.6514 | 0.057 | |
| 2F | spine head polyribosomes | 20.49 | 0.0000 | 0.255 | 1.13 | 0.3591 | 0.116 | 4.23 | 0.0461 | 0.094 | 2.39 | 0.0660 | 0.189 | |
| 2H | filopodia with PR | 6.61 | 0.0126 | 0.099 | 0.43 | 0.8779 | 0.048 | 1.70 | 0.1999 | 0.040 | 1.00 | 0.4211 | 0.088 | |
| 2H | filopodia without PR | 3.72 | 0.0584 | 0.058 | 0.73 | 0.6433 | 0.079 | 12.02 | 0.0012 | 0.227 | 0.82 | 0.5173 | 0.074 | |
| 3D | spines without PR, PSD < 0.05 μm^2^ | 1.12 | 0.2933 | 0.018 | 1.74 | 0.1163 | 0.169 | 5.65 | 0.0222 | 0.121 | 1.65 | 0.1798 | 0.139 | |
| 3E | spines with PR, PSD < 0.05 μm^2^ | 5.93 | 0.0179 | 0.090 | 1.16 | 0.3402 | 0.119 | 0.01 | 0.9436 | 0.000 | 2.28 | 0.0773 | 0.182 | |
| 3E | spines with PR, PSD < 0.1 μm^2^ | 6.58 | 0.0128 | 0.099 | 1.21 | 0.3112 | 0.124 | 1.14 | 0.2922 | 0.027 | 2.33 | 0.0719 | 0.185 | |
| 3E | spines with PR, PSD > 0.2 μm^2^ | 10.61 | 0.0019 | 0.150 | 1.37 | 0.2342 | 0.138 | 0.75 | 0.3914 | 0.018 | 0.91 | 0.4684 | 0.081 | |
| 3F | spines with base PR, PSD < 0.1 μm^2^ | 8.12 | 0.0060 | 0.119 | 0.98 | 0.4524 | 0.103 | 0.64 | 0.4273 | 0.015 | 0.86 | 0.4939 | 0.078 | |
| 3F | spines with base PR, PSD > 0.2 μm^2^ | 5.83 | 0.0188 | 0.089 | 1.48 | 0.1919 | 0.147 | 0.38 | 0.5399 | 0.009 | 1.00 | 0.4200 | 0.089 | |
| 3H | spines with head PR, PSD < 0.05 μm^2^ | 10.63 | 0.0018 | 0.150 | 0.62 | 0.7337 | 0.068 | 4.54 | 0.0391 | 0.100 | 2.74 | 0.0414 | 0.211 | |
| 3H | spines with head PR, PSD < 0.1 μm^2^ | 10.33 | 0.0021 | 0.147 | 1.46 | 0.1984 | 0.146 | 0.04 | 0.8424 | 0.001 | 1.41 | 0.2492 | 0.121 | |
| 3H | spines with head PR, PSD < 0.15 μm^2^ | 6.55 | 0.0130 | 0.098 | 2.38 | 0.0322 | 0.218 | 1.62 | 0.2107 | 0.038 | 0.13 | 0.9721 | 0.012 | |
| 3H | spines with head PR, PSD > 0.2 μm^2^ | 7.87 | 0.0068 | 0.116 | 0.97 | 0.4645 | 0.101 | 1.51 | 0.2260 | 0.036 | 0.45 | 0.7708 | 0.042 | |
| 4B | spines with spine apparatus, with PR | 12.63 | 0.0007 | 0.174 | 1.18 | 0.3252 | 0.121 | 0.00 | 0.9532 | 0.000 | 0.90 | 0.4709 | 0.081 | |
| 4B | spines with spine apparatus, no PR | 0.28 | 0.6015 | 0.005 | 1.81 | 0.1016 | 0.174 | 2.78 | 0.1032 | 0.063 | 3.02 | 0.0286 | 0.227 | |
| 4C | spines without spine apparatus, with PR | 5.79 | 0.0192 | 0.088 | 0.90 | 0.5159 | 0.095 | 0.06 | 0.8072 | 0.001 | 2.22 | 0.0834 | 0.178 | |
| 4C | spines without spine apparatus, no PR | 1.38 | 0.2443 | 0.023 | 1.91 | 0.0838 | 0.182 | 4.67 | 0.0366 | 0.102 | 1.34 | 0.2727 | 0.115 | |
| 5B | shaft synapses | 0.02 | 0.8808 | 0.000 | 0.73 | 0.6450 | 0.079 | 4.79 | 0.0344 | 0.105 | 1.52 | 0.2145 | 0.129 | |
| 5C | shaft synapses, PSD < 0.1 μm^2^ | 0.26 | 0.6151 | 0.004 | 1.43 | 0.2105 | 0.143 | 4.81 | 0.0340 | 0.105 | 1.07 | 0.3837 | 0.095 | |
|  |  |  |  |  |  |  |  |  |  |  |  |  |  | |
